# Transactions of the Medical Society of Tennessee

**Published:** 1883-08-20

**Authors:** 


### *TRANSACTIONS OF THE MEDICAL SOCIETY OF TENNESSEE.*

ASSEMBLED AT NASHVILLE, APRIL, 1884.

It contains 104 pages, neatly printed.

The address of welcome was made by Deering J. Roberts, M.D. Also, an address of welcome by Gov. Bates.

Annual Address by W. F. Glenn, M.D., the President—subject, "Is Man Immortal?"—followed by the following interesting papers:

Historical reminiscences, by Thos. Lipscomb, M.D.
Perils of Femoral Hernia. Case reported—Enteroraphy, by A. B. Tadlock, M.D.
Plastic Surgery, by J G Sinclair, M D.
Ovarian Tumor of Twenty-two Years Duration, by W D Haygood, M D.
Vaccination and Smallpox, by J S Nowlen M D.
Case of Induced Delivery, by J W Davis, M D.
Obituary of Dr. Chas K Winston, by W P Jones, M D.
Obituary of Walter H. Sims, M D, by Thos Lipscomb, M D.
Also, Obituary notice of Rudolph Knaffle, M D.

We have not space now to notice the papers above-mentioned, some of which contain interesting matter for future reference.

